# Influence of soil spatial variability on the reliability of sandy slopes incorporating anisotropy and non-stationarity

**DOI:** 10.1371/journal.pone.0323471

**Published:** 2025-05-15

**Authors:** Li-Qun Zheng, Yuan Zhou, Lei Huang, Ling-Jun Huang, Xiao-Qiang Fu

**Affiliations:** 1 Fujian No. 1 Construction Group Co., Ltd., Fujian, China; 2 College of Architecture and Civil Engineering, Sanming University, Sanming, China; 3 Key Laboratory of Intelligent Construction and Monitoring of Engineering Structures in Fujian Province College, Sanming, China; China University of Mining and Technology, CHINA

## Abstract

Influence of soil spatial variability on the reliability of cohesive soil slopes has been investigated extensively in the previous studies. However, it is seldom investigated for sandy slopes, especially incorporating anisotropy and non-stationarity. This paper explores the impact of the anisotropic spatial variation and non-stationary characteristics of soils on the reliability of sandy slopes. Specifically, two types of non-stationary random field (RF) are included, namely RF Type 1(soil strength increases along the depth) and RF Type 2 (strength increases along the direction perpendicular to bedding). For sandy slopes, the reliability index (*β*) is stable as the major autocorrelation distance of soil properties increases, and it decreases as the minor autocorrelation distance increases, which aligns with the observations for clay slopes. For slopes with horizontal bedding, the results of *β* under stationary RF are close to those under RF type 1. For dip-slopes and reverse-dip slopes with a 1:1 ratio, the difference between *β* under stationary RF and RF Type 1 is small. For dip-slopes with 1:2 ratio, this difference remains small, but for the reverse-dip slopes, it is significant. These findings differ from the previous studies on clay slopes, where the difference between *β* under stationary RF and RF Type 1 is significant for each slope scenario. Besides, *β* under RF type 2 is the smallest for dip slopes and reverse-dip slopes with ratio = 1:2, and it is the highest for reverse-dip slopes with ratio = 1:1.

## Introduction

Soil spatial variability involving anisotropy and non-stationarity is a common and significant phenomenon in natural soils. Anisotropy in soils can be manifested in different forms, such as horizontal transverse anisotropy and rotated transverse anisotropy [1–3]. Meanwhile, non-stationarity of soils means that a soil property fluctuates around a trend which usually increases with depth [4]. In such a situation, the fluctuating part is considered as the inherent variability of the soil and the trend function represents the mean value of the soil property at different depths. Here, a number of in-situ test data have shown that soil properties in a statistically homogeneous soil layer can display non-constant trends along the depth [5–9]. The combination of anisotropy and non-stationarity in soil properties presents two distinct scenarios in terms of the directions of the increasing trend [10]. In the first scenario, denoted as RF type 1, the strength of the soil has a tendency to increase with depth. This typically happens on soil strata that were originally inclined. That is to say, the accumulation of soil was inclined from the start. For instance, at the edge of a sedimentary basin, the soils are often deposited at an oblique angle. As for the second scenario, named RF type 2, the soil property shows an increasing trend along the direction perpendicular to the bedding. This can be observed in rotated strata which are formed due to tectonic movements or deltaic deposits. For example, the soil bedding was originally in a horizontal position and becomes rotated because of tectonic activities. Both the anisotropic spatial variation of soils and the non-stationary characteristics are widely existing features in natural soils. Thus, it is of great importance to study their combined influence on the stability of slopes. Previously, Huang et al. [10] investigated the combined influence of the anisotropic spatial variation of undrained soil strength and the non-stationary characteristics on the reliability of saturated clay slopes. However, to the best of our knowledge, there has been no research discussing their combined influence on sandy slopes.

In the existing body of research, a large number of studies have delved into the influence of soil spatial variability on the reliability of slopes. Among these, the random field (RF) theory has been widely adopted to simulate soil spatial variability [1,11–24]. For instance, Cho [11] used the random limit equilibrium method (RLEM), which combines the limit equilibrium method and random field theory, to investigate the influence of the cross-correlation between cohesion (*c*) and friction angle (*φ*) on the failure probability of slopes under drained conditions. Huang and Leung [1] explored the impact of rotated transverse anisotropy in soil properties on the reliability of slopes under undrained conditions, using three-dimensional anisotropic random field models. Li et al. [17] established a random field model considering the non-stationary characteristics of soil. They respectively studied the influence of the non-stationary characteristics of soil on the reliability of saturated clay slopes under undrained conditions and the impact of the non-stationary characteristics of the friction angle on sandy slopes. Based on the previous fruitful studies, the geotechnical profession has already gained a relatively profound understanding of the influence of soil spatial variability on the reliability of cohesive soil slopes. For sandy soils, the shear strength mainly depends on the friction angle, while that of cohesive soil relies on the combined effect of cohesion and friction angle. Due to the lack of cohesion in sandy soils, the stability of a sandy slope is more susceptible to local fluctuations in the spatial distribution of shear strength (e.g., the concentration of weak zones caused by heterogeneity), whereas cohesive soil has a stronger “buffering” capacity against local variability due to the presence of cohesion. Nevertheless, there are fewer relevant studies focusing on sandy slopes. In particular, the interplay between rotated transverse anisotropy and non-stationarity has not yet been explored for sandy slopes.

The aim of this study is to comprehensively analyze the influence of the spatial anisotropy and non-stationary characteristics of soils on the reliability of sandy slopes. By comparing the values of reliability index (*β*) in various slopes with horizontal bedding and inclined bedding under stationary RF, RF types 1 and 2, this study identifies scenarios where neglecting non-stationarity leads to significant changes of slope reliability. To this end, the current study adopts the random limit equilibrium method and combines the anisotropic and non-stationary random field theories in the reliability assessment of sandy slopes. This paper is structured into four main parts. Firstly, the “Introduction” section mainly discusses the background of soil spatial variability, anisotropic spatial variation of soils, and the non-stationary characteristics. It also provides a brief review of relevant studies on the influence of these factors on the reliability of slopes. Secondly, the “Methodology” section elaborates on how to establish the random field models considering anisotropic spatial variation of soils and the non-stationarity. Thirdly, the “Results” section presents the analytical results of this study. Finally, the “Conclusion” section summarizes and discusses the main findings.

## Methodology

The spatial variability of the friction angle in sandy soil is mainly simulated based on the random field theory [25] in this study. In this process, two different random field models are adopted: the stationary random field model and the non-stationary random field model. For the stationary random field model, it is characterized that its trend structure is represented as a constant value, which means that the mean value of soil parameters is kept the same throughout the entire spatial domain. By contrast, in the non-stationary random field model, the mean value of parameters is shown to have a tendency to increase gradually with depth. Furthermore, the random limit equilibrium method is adopted to compute the reliability of slopes in this study, and the implementation process of this method is also introduced in this section.

### Modelling of soil spatial variability

The soil in nature has the characteristic of spatial variability. In general, the soil parameters with spatial variability are usually expressed by the following formula:


$${\text{z}}_{j}^{\left(i\right)}={\;\;\;\mu \;\;\;}_{j}+\sqrt{\sigma^{2}}\varepsilon_{j}^{\left(i\right)}$$ (1)

where the parameter $z_{j}^{\left(i\right)}$ represents a spatially variable soil parameter at location *j* that corresponds to the *i*th realization; $\mu_{j}$ stands for the value of the trend of the soil parameter at location *j*; *σ* denotes the standard deviation of the spatially variable soil parameter; and $\varepsilon_{j}^{\left(i\right)}$ refers to an element in the *i*th realization of the standard Gaussian random field at location *j*. The **ε** can be acquired through the use of random field models, which are elaborated in the following subsections.

### Modelling of stationary random field (RF)

When simulating stationary random field, $\mu_{j}$ in Eq. (1) is taken as a constant number, while the **ε** is generated using random field. In the literature, there are various random field generation techniques that can be used to obtain **ε** in Eq. (1), such as, Cholesky decomposition [26], local average subdivision method [27], and Karhunen-Loeve expansion method [28]. In this study, the Cholesky decomposition method is adopted to generate random field variables, because it is easier to operate when dealing with the anisotropic random fields:


$$\text{R}=LL^{T}$$ (2)

$$\varepsilon =L{\text{s}}_{i}$$ (3)

In the above equations, **R** denotes the spatial autocorrelation matrix; **L** denotes the Cholesky factor of **R**; **s**_*i*_ signifies a vector containing independent standard Gaussian numbers associated with the *i*-th realization of the random field. The matrix **R** can be derived as follows:


$$\begin{array}{c} {𝐑}_{d\times d}=\left[\begin{array}{cccc} 1 & \rho_{12} & \cdots & \rho_{1d}\\ \rho_{21} & 1 & \cdots & \rho_{2d}\\ \vdots & \vdots & \ddots & \vdots \\ \rho_{d1} & \rho_{d2} & \cdots & 1 \end{array}\right] \end{array}$$ (4)

where the symbol *d* stands for the quantity of elements within the random field. Meanwhile, *ρ*_12_ represents the spatial correlation coefficient between positions 1 and 2. The correlation coefficient can be obtained via the autocorrelation function as follows, where the rotation of random field is incorporated in the function to simulate the slope with inclined bedding:


$$\rho (\Delta_{x},\Delta_{y})=\exp\left[-\left(\frac{{\left(\Delta_{x}\cos\alpha +\Delta_{y}\sin\alpha \right)}^{2}}{{\theta_{1}}^{2}}+\frac{{\left(-\Delta_{x}\sin\alpha +\Delta_{y}\cos\alpha \right)}^{2}}{{\theta_{2}}^{2}}\right)\right]$$ (5)

where Δ_*x*_ and Δ_*y*_ represent the lag between any two positions in the *x* and *y* directions, respectively; *θ*_1_ and *θ*_2_ represent the major and minor autocorrelation distances, respectively; and *α* denotes the angle of rotation of the random fields, which also indicates the dip angle of soil bedding. Here, *α > *0 indicates anticlockwise rotation, and *α < *0 indicates clockwise rotation.

### Modelling of non-stationary random field

The non-stationary random field model of friction angle was proposed by Li et al. [17], which is derived based on the observation by Phoon and Kulhawy [4]. Meanwhile, the accuracy of the model is checked by comparing the simulated results with the CPT data in Li et al. [17]. Here, the spatial variation of the friction angle can be decomposed into a smoothly varying trend function *t*(*z*) and a fluctuating component ***w***(*j*), as described below:


ϕ(j)=t(z)+w(j) (6)

where *φ*(*j*) represents the value of friction angle at location *j*; *t*(z) represents the value of the trend at depth *z*; and the fluctuating component ***w***(*j*) can be generated using stationary random field through Eqs. (1) - (5). Here, the trend function *t*(*z*) can be expressed by the following equation:


t(z)=az+b (7)

where *a* denotes the change rate of the mean with depth *z*; and *b* represents the mean value at the ground surface (i.e., *z* = 0).

It should be noted that in the current work, two types of non-stationary RF are incorporated. For RF type 1, the soil strength has an increasing tendency along the depth, and the non-stationary RF can be simulated directly trough Eqs. (1) - (6). For RF type 2, the soil strength increases along the direction perpendicular to the bedding. In this case, the non-stationary RF can be realized by rotating the non-stationary RF associated with horizontal bedding, and the equations for the trend function *t*(*z*) can be expressed as following Eqs. (8) and (9) in relation to clockwise and anti-clockwise rotation, respectively [10]:


t(z)=b+a[(W−x)sinα+zcosα] (8)

t(z)=b+a(xsinα+zcosα) (9)

where *W* denotes the width of the slope model;

### Implementation procedures of random limit equilibrium method (RLEM)

In the current work, random limit equilibrium method is adopted to compute the reliability index of slopes. In the context of RLEM, the soil property distributed in the problem domain is simulated by a random field, and the factor of safety (FS) of a slope is then computed by limit equilibrium method. Monte Carlo simulation (MCS) associated with a number of random field realizations is conducted to determine the statistical characteristics of FS (i.e., mean and standard deviation of FS). Subsequently, the reliability index of the slope can be obtained through the following equation, which assumes the FS follows log-normal distribution:


$$\beta =\frac{ln\left[\frac{\mu_{FS}}{\sqrt{1+{V_{FS}}^{2}}}\right]}{\sqrt{ln[1+{V_{FS}}^{2}]}}$$ (10)

In the above equation, *μ*_FS_ denotes the mean value of FS; *V*_FS_ = *μ*_FS_/ *σ*_FS_, where *σ*_FS_ represents the standard deviation of FS; and *β* represents the slope reliability index.

**Fig 1:**
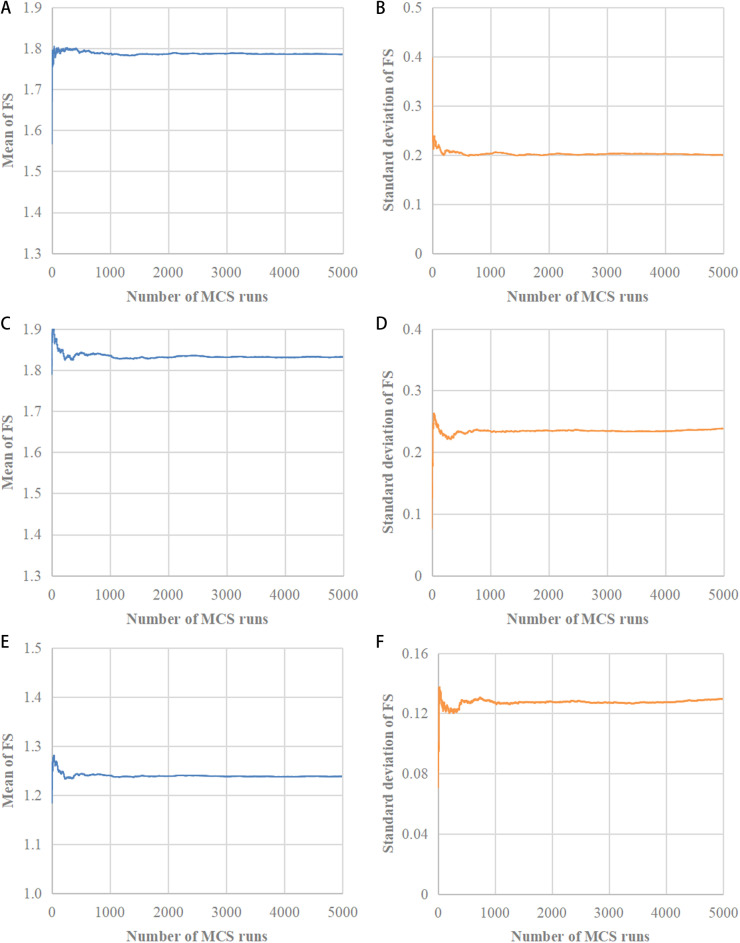
Change of mean of FS,*μ*_FS_**, and standard deviation of FS,** *σ*_FS_**, with the number of Monte Carlo simulation runs for slopes with** *α = *45°, *θ*_1 _= 20 m and *θ*_2_ = 2 m. **(a)** Change of *μ*_FS_ under stationary RF. **(b)** Change of *σ*_FS_ under stationary RF. **(c)** Change of *μ*_FS_ under RF type 1. **(d)** Change of *σ*_FS_ under RF type 1. **(e)** Change of *μ*_FS_ under RF type 1. **(f)** Change of *σ*_FS_ under RF type 1.

The implementation procedures of RLEM used in the current work are presented as follows:

Step 1: Generation of random fields. RFs (random fields) are generated with *N* realizations. For stationary RF, Eqs. (1) - (5) are utilized, while for non-stationary RFs, Eqs. (6) - (9) are employed. In the present work, 1000 realizations are taken into account (equivalent to 1000 Monte Carlo simulation runs). 1000 realizations are adequate for determining *μ*_FS_ and *σ*_FS_ as reported by a number of previous studies [29–33]. Also, the adequacy of the use of 1000 realizations is validated in Fig. 1.

Step 2: Setting up the slope stability analysis model. In the present work, the Slope/W software package [34] is employed to establish the slope stability analysis model. For slope stability analysis, the Bishop method is adopted, because the computation efficiency of this method is relatively high under the framework of MCS. Also, the convergence issue is insignificant compared with other limit equilibrium methods when considering random fields [32].

Step 3: Combining the RFs and the slope stability analysis model. The RFs are output as “.txt” files, while the slope stability analysis model is output as “.xml” files. After that, these two types of files are combined to generate *N* “.xml” files which incorporate the information of the slope stability analysis model along with the RFs.

Step 4: Conducting Monte Carlo simulation. The *N* “.xml” files produced in Step 3 are executed by batch processing to calculate *N* values of FS. Then, statistical analyses are carried out on these outputs to obtain *μ*_FS_ and *σ*_FS_. Subsequently, the reliability index is determined through Eq. (10).

## Results

This section firstly describes the illustrative examples adopted in this study and the parameter settings. Subsequently, it shows how the spatial variability and non-stationary characteristics of soils affect the reliability index of sandy slopes, considering the horizontal transverse anisotropy and rotated transverse anisotropy respectively.

### Illustrative example

**Fig 2:**
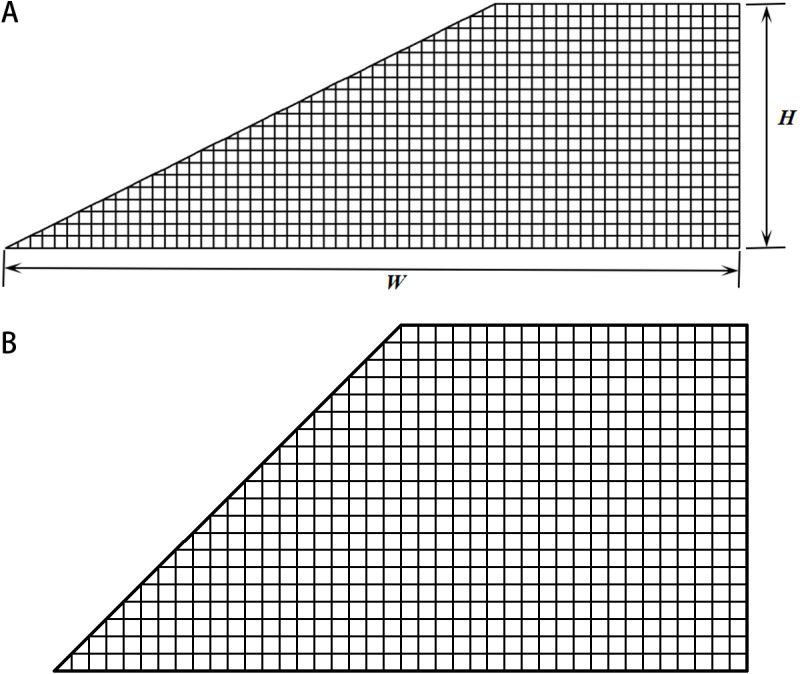
The slope models investigated in the current work. **(a)** Model 1, slope ratio = 1:2. **(b)** Model 2, slope ratio = 1:1.

In the current work, models with two different slope ratios are incorporated (Fig 2), as the slope ratio can affect the potential depth of slip surface [35] and thus the slope performance under various scenarios of random fields. The heights of the slope models (*H*) are 10 m, and the widths are 30 m and 20 m for models 1 and 2, respectively. The sandy slopes are under drained conditions, where the cohesion values of the slopes are set to constant values equal to 2 kPa and the friction angles are set to random field variables. For the parameters of the stationary RF, the mean of the friction angle, *μ*_*φ*_, is set to 38.5° and the corresponding coefficient of variation (*CoV*_*φ*_) is set to 0.15. For the parameters of non-stationary RF, *a* and *b* in Eq. 7 are set to 1.4 °/m and 31.5°, respectively, which ensures that the value of friction angle at the mid-depth is equal to 38.5°. These settings are referred to Li et al. [17]. The autocorrelation distances and rotational angles of soil bedding are varied. Here, the major autocorrelation distance, *θ*_*1*_, varies in {15 m, 20 m, 25 m, 30 m, 35 m, 40 m}; the minor autocorrelation distance, *θ*_*2*_, varies in {1 m, 1.5 m, 2 m, 2.5 m, 3 m, 3.5 m, 4 m}; and the rotational angle of soil bedding, *α*, varies in {-15°, -30°, -45°, -60°, -75°, 15°, 30°, 45°, 60°, 75°}. The discretization of the random fields is shown in Fig 2, where the sizes of the square elements are equal to 0.5 m.

### Results associated with horizontal transverse anisotropy

**Fig 3:**
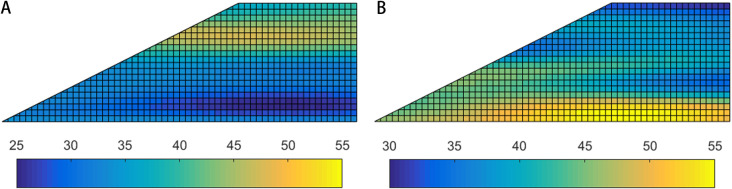
Typical realizations of random fields of friction angle (*φ*) associated with horizontal transverse anisotropy (i.e., rotational angle *α = *0°). **(a)** Stationary RF, *θ*_*1 *_= 20 m, *θ*_*2*_ = 2 m and *α = *0°.(b) Non-stationary RF, *θ*_*1 *_= 20 m, *θ*_*2*_ = 2 m and *α = *0°.

Horizontal transverse anisotropy of soils can be observed in natural slopes with horizontal stratification, which can be simulated by anisotropic random fields incorporating Eq. (5). Fig 3 shows the typical realizations of anisotropic random fields of friction angle associated with horizontal transverse anisotropy of soils.

**Fig 4:**
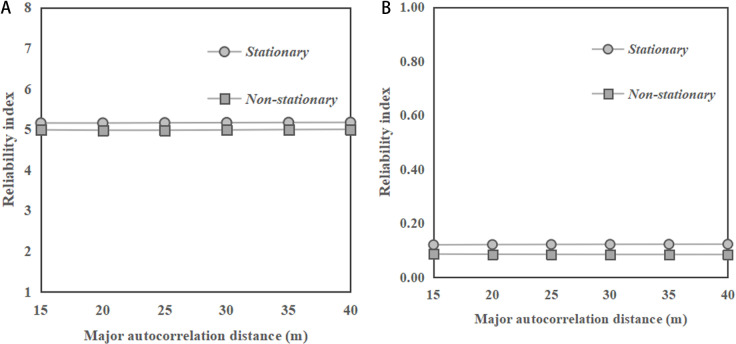
Change of reliability indices of the sandy slopes with major autocorrelation distance of *φ* for horizontal transverse anisotropy of soils. **(a)** Slope ratio = 1:2. **(b)** Slope ratio = 1:1.

Figs 4(a) and (b) illustrate the change of reliability indices of the sandy slopes with major autocorrelation distance of *φ* under horizontal transverse anisotropy of soils for slope ratios of 1:2 and 1:1, respectively. In general, the reliability index shows a stable trend as the major autocorrelation distance increases, under stationary and non-stationary RFs for both slope ratios. Such an observation is consistent with the findings reported by the previous studies for clay slopes [1,14]. In addition, it can be found from the figures that the results obtained under non-stationary RF are slightly smaller than those obtained under stationary RF. This observation is different from that in the cases of clay slopes under undrained conditions, where the results of reliability index obtained under the non-stationary RF of undrained soil strength are significantly larger than those obtained under stationary RF, as reported by Huang et al. [10]. Moreover, it can be noted from the figures that the reliability indices of the slope with ratio of 1:1 are significantly smaller than those with ratio of 1:2.

**Fig 5:**
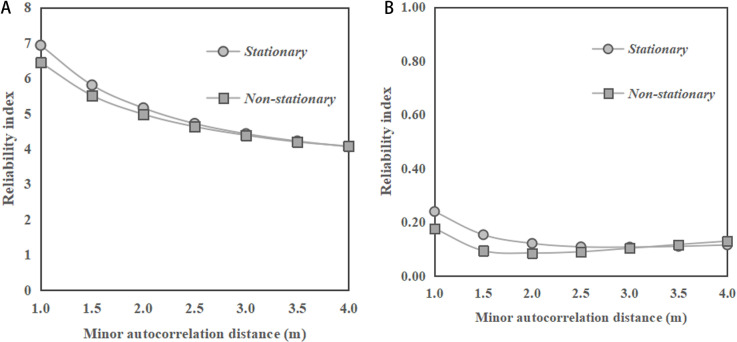
Change of reliability indices of the sandy slopes with minor autocorrelation distance of *φ* for horizontal transverse anisotropy of soils. **(a)** Slope ratio = 1:2. **(b)** Slope ratio = 1:1.

Fig 5 depicts the change of reliability indices of sandy slopes with minor autocorrelation distance for horizontal transverse anisotropy of soils. In Fig 5(a), with a slope ratio of 1:2, the reliability index values for both stationary and non - stationary RFs show a significant decreasing trend as the minor autocorrelation distance increases. Besides, it can be observed that the reliability index for the stationary RF is slightly higher than that for the non-stationary RF across the entire range of minor autocorrelation distances considered. In Fig 5(b), with a slope ratio of 1:1, the reliability indices for both stationary and non-stationary conditions generally decrease as the minor autocorrelation distance increases. Similar to the case with slope ratio of 1:2, in Fig 5(a), the stationary condition has a slightly higher reliability index than those under non-stationary RF. These observations are different from the previous observations for the cases of saturated clay slope under undrained conditions, where the results of reliability index obtained under the non-stationary RF of undrained soil strength are significantly larger than those obtained under stationary RF, as reported by Huang et al. [10]. When comparing the two slope ratios, it is observed that the reliability indices for both stationary and non-stationary conditions are higher in Fig 5(a) with a slope ratio of 1:2 than in Fig 5(b) with a slope ratio of 1:1.

In summary, for horizontal transverse anisotropy of soils, the reliability index has a stable trend as the major autocorrelation distance increases under both stationary and non-stationary RFs. Meanwhile, the results of the reliability index under both stationary and non-stationary RFs show a decreasing trend in reliability indices as the minor autocorrelation distance increases. These observations are consistent with the findings reported in the previous studies related to clay slopes. However, the results under non-stationary RF are slightly smaller than the results under stationary RF, which is different from the case of saturated clay slopes under undrained conditions.

### Results associated with rotated transverse anisotropy

**Fig 6:**
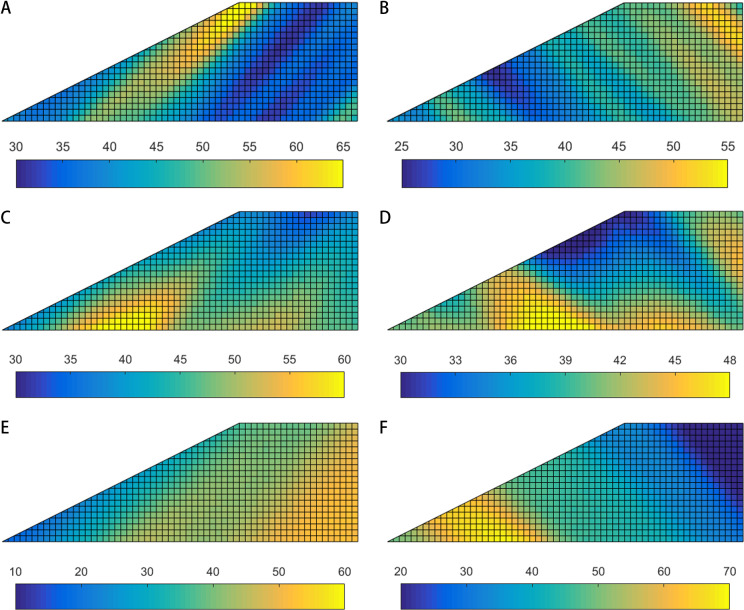
Typical realizations of random fields of friction angle associated with rotated transverse anisotropy. **(a)** Stationary RF for dip slope, *θ*_*1 *_= 20 m, *θ*_*2*_ = 2 m and *α = *45°. **(b)** Stationary RF for reverse-dip slope, *θ*_*1 *_= 20 m, *θ*_*2*_ = 2 m and *α = -* 45°. **(c)** RF type 1 for dip slope, *θ*_*1 *_= 20 m, *θ*_*2*_ = 3 m and *α = *45°. **(d)** RF type 1 for reverse-dip slope, *θ*_*1 *_= 20 m, *θ*_*2*_ = 3 m and *α = -*45°. **(e)** RF type 2 for dip slope, *θ*_*1 *_= 20 m, *θ*_*2*_ = 3 m and *α = *45°. **(f)** RF type 2 for reverse-dip slope, *θ*_*1 *_= 20 m, *θ*_*2*_ = 3 m and *α = -*45°.

Rotated transverse anisotropy can be observed in natural slopes with oblique stratification. In general, there are two types of obliquely bedded slopes, i.e., dip slope and reverse-dip slope. For a dip slope, the dip direction of the bedding is the same as that of the slope, while for a reverse-dip slope, the dip direction of the bedding is opposite to the dip direction of the slope. In addition, two types of non-stationary RF are incorporated in the current work, where the soil strength increases along depth (RF type 1) and the direction perpendicular to bedding (RF type 2), respectively, as discussed in the “Introduction”. Fig 6 shows the typical realizations of anisotropic random fields of friction angle associated with rotated transverse anisotropy of soils.

**Fig 7:**
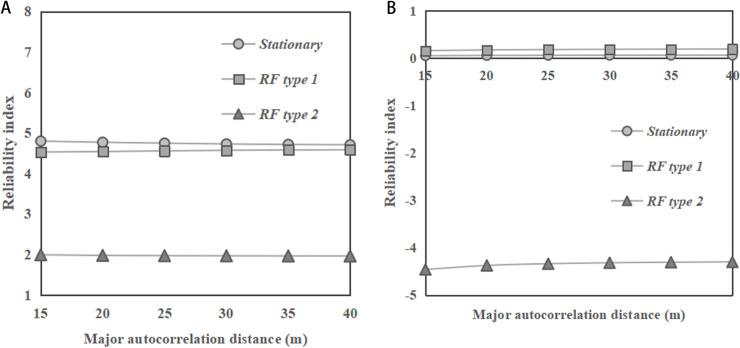
Change of reliability indices of the dip slopes (*α = *45°) with major autocorrelation distance of *φ*. **(a)** Slope ratio = 1:2. **(b)** Slope ratio = 1:1.

Fig 7 illustrates the change of reliability indices of the dip slopes with the major autocorrelation distance of *φ*. In Figs 7(a) and (b), the reliability index values show a stable pattern with the change of the major autocorrelation distance for various types of RF and slope ratios. These observations are consistent with those for the horizontal transverse anisotropy of soils. In addition, as shown in Fig 7, the stationary RF has a significantly higher reliability index compared to RF type 2, while RF type 1 has a reliability index close to that under stationary RF for both slope ratios. These findings are different from the previous observations for the cases of clay dip-slopes, where the results of reliability index obtained under the stationary RF and RF type 1 of undrained soil strength are significant different, as reported by Huang et al. [10].

**Fig 8:**
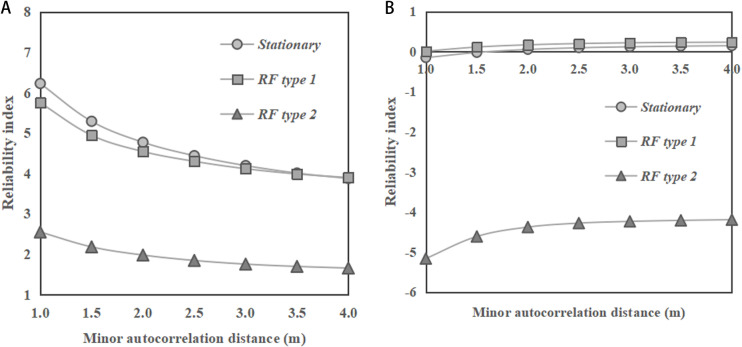
Change of reliability indices of the dip slopes (*α = *45°) with minor autocorrelation distance of *φ*. **(a)** Slope ratio = 1:2. **(b)** Slope ratio = 1:1.

Fig 8 shows the change of reliability indices of the dip slopes with the minor autocorrelation distance of *φ*. In Fig 8(a) with a slope ratio of 1:2, the reliability index values show a decreasing trend for all RF types (stationary RF, RF type 1, and RF type 2) with the minor autocorrelation distance, while in Fig 8(b) with a slope ratio of 1:1, the reliability index values show a slightly increasing trend. Besides, it can be observed from the figures that the RF type 2 has the smallest reliability index values among the three RF types, while the results obtained under stationary RF and RF type 1 are close. These findings are also different from the previous observations for the cases of saturated clay dip slopes, where the results of reliability index obtained under the stationary RF and RF type 1 of undrained soil strength are significant different, as reported by Huang et al. [10]. Comparing Figs 8(a) and (b), the slope ratio of 1:2 generally shows significantly higher reliability index values across all types (stationary RF, RF type 1, and RF type 2) compared to the slope ratio of 1:1.

**Fig 9:**
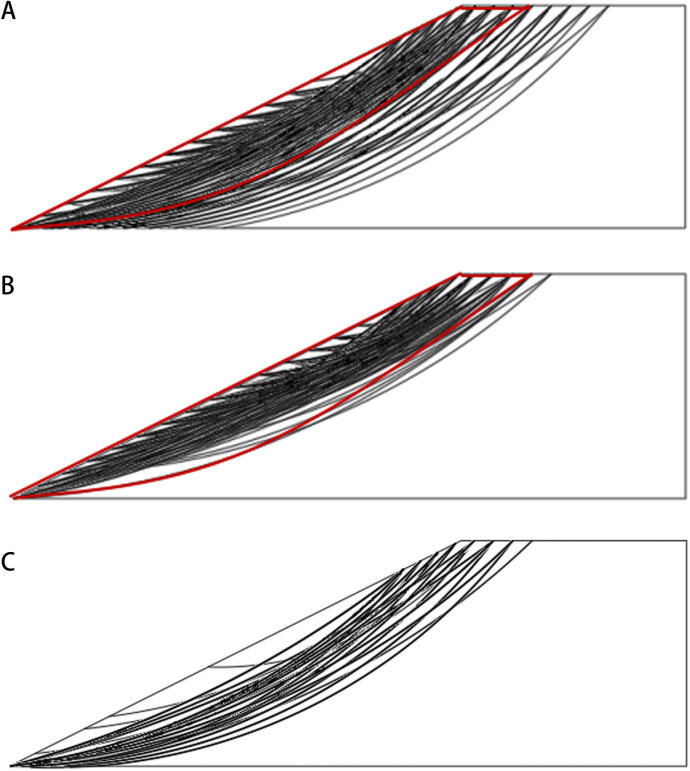
The distribution of slip surfaces obtained by 1000 MCSs: (a) stationary RF;**(b)** RF type 1; and **(c)** RF type 2.

Fig 9 shows the distribution of slip surfaces obtained by 1000 MCSs under various RF types. It can be observed from the figures that under stationary RF and RF type 1, the main regions where the slip surfaces are concentrated are roughly the same, and the distribution patterns of slip surfaces are more discrete than those under RF type 2. Here, under RF type 2, the slope fails roughly along the weak bedding plane, while under stationary RF and RF type 1, the slip surface will pass through more bedding planes with higher strength, which makes the average value of FS under RF type 2 smaller. Therefore, the reliability indices under the stationary RF and RF type 1 are close, while the reliability index under RF type 2 is smaller, as shown in Figs 7 and 8. It means that when designing a dip slope with sandy soils, the investigation resources for the non-stationary characteristics of soils can be reduced if the soil strength increases with depth, while investigation resources are crucial if the soil strength increases along the direction perpendicular to bedding.

**Fig 10:**
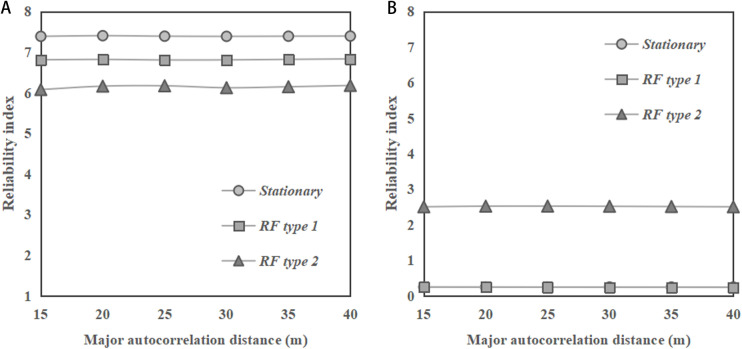
Change of reliability indices of the reverse-dip slopes (*α = -*45°) with major autocorrelation distance of *φ*. **(a)** Slope ratio = 1:2. **(b)** Slope ratio = 1:1.

Figs 10(a) and (b) show the change of reliability indices of the reverse-dip slopes with the major autocorrelation distance of *φ*, for slope ratios of 1:2 and 1:1, respectively. It can be found from the figures that the reliability index values show a stable pattern with the change of the major autocorrelation distance for various types of RF and slope ratios. These observations are consistent with those for horizontal transverse anisotropy and the dip slope. In Fig 10(a), the stationary RF has a higher reliability index compared to RF type 2, while RF type 1 has a reliability index in between. The difference between results of reliability index under various RF types is significant, and this observation is different from that under dip-slopes, where the results obtained under stationary RF and RF type 1 are close, as indicated in Fig 10. It is interesting to find that in Fig 10(b), the RF type 2 has a highest reliability index, while the results under stationary RF and RF type 1 are close. These findings are also different from those observed under the cases of saturated clay slope under undrained conditions. Here, for the reverse-dip clay slope under undrained conditions, the difference between the reliability indices obtained under stationary RF and RF type 1 were significant, as reported by Huang et al. [10]. Comparing Figs. 10 (a) and (b), the slope ratio of 1:2 generally shows higher reliability index values across all types (stationary RF, RF type 1 and RF type 2) compared to the slope ratio of 1:1.

**Fig 11:**
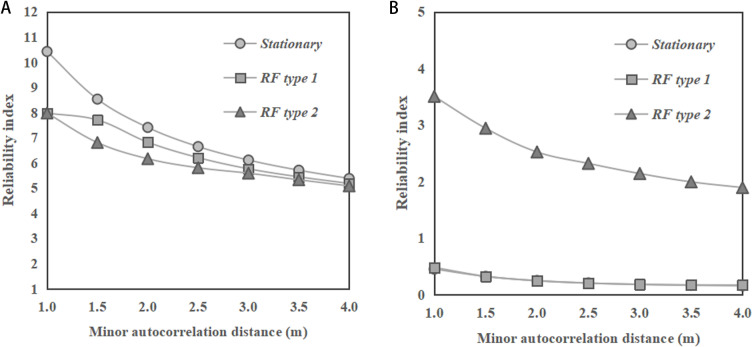
Change of reliability indices of the reverse-dip slopes (*α = -*45°) with minor autocorrelation distance of *φ*. **(a)** Slope ratio = 1:2. **(b)** Slope ratio = 1:1.

Fig 11 shows the change of reliability indices of the reverse-dip slopes with the minor autocorrelation distance of *φ*. In Figs 11(a) and (b) with slope ratios of 1:2 and 1:1, the reliability index values show a decreasing trend for all types (stationary RF, RF type 1, and RF type 2) with the minor autocorrelation distance. It can be observed from Fig 11(a) that the stationary RF has a higher reliability index compared to RF type 2, while RF type 1 has a reliability index in between. The difference between results of reliability index under various RF types is significant, and this observation is different from that under dip-slopes, where the results obtained under stationary RF and RF type 1 are close, as indicated in Fig 11. It can also be found that in Fig 11(b), the RF type 2 has a highest reliability index, while the results under stationary RF and RF type 1 are close. Comparing Figs 11(a) and (b), the slope ratio of 1:2 generally shows significantly higher reliability index values across all types (stationary RF, RF type 1, and RF type 2) compared to the slope ratio of 1:1.

**Fig 12:**
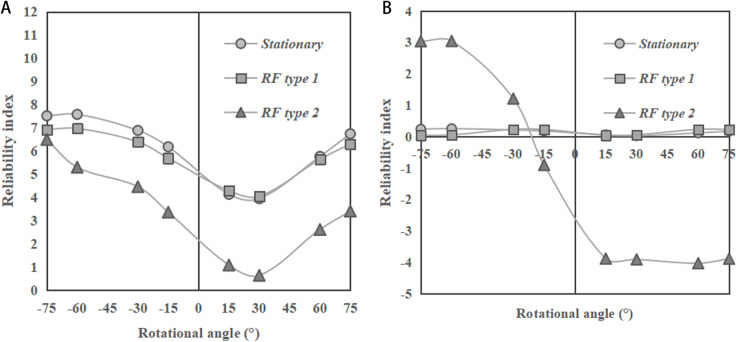
Change of reliability indices of the sandy slopes with rotational angle of bedding (*α > *0 indicates anticlockwise rotation, and *α < *0 indicates clockwise rotation). **(a)** Slope ratio = 1:2. **(b)** Slope ratio = 1:1.

Figs 12(a) and (b) show the variations of reliability index with the rotational angles of bedding for slopes with ratios of 1:2 and 1:1, respectively. In general, it can be seen that the reliability index of the slope with ratio of 1:2 is larger than that with ratio of 1:1 at each rotational angle. For a given slope ratio, the reliability indices under rotational angle smaller than 0 (indicating reverse-dip slopes) are generally larger than those under rotational angle larger than 0 (indicating dip slopes). That is because in a dip slope, the slip surface is easier to form due to the impact from the weak bedding plane, while in a reverse-dip slope, the slip surface is more difficult to form, as it should pass through multiple bedding planes with various soil strength. This observation is consistent with those found in the previous studies for cohesive soil slopes, reported by Zhu et al. [3] and Huang et al. [10].

In addition, it can be found from the figures that the results under stationary RF are close to those under RF type 1, for the slope ratio of 1:2 with rotational angle < 0 and the slope ratio of 1:1 with all the rotational angles. For the slope ratio of 1:2 with rotational angle < 0 (i.e., reverse-dip slopes), the difference between the values of reliability index under stationary RF and RF type 1 is significant. This observation echoes those covered in the previous paragraph, as indicated in Figs 7 - 11. This is because in a gentler slope (slope ratio = 1:2), slip surfaces typically develop deeper into the soil mass [35]. Meanwhile, for the reverse dip slopes, the potential slip surfaces will pass through multiple bedding planes, meaning that the bedding direction has a smaller influence on the path of slip surface. In this situation, for stationary RF, where the soil strength is randomly distributed without a trend, deeper slip surfaces are likely to form. By contrast, for RF type 1, slip surfaces tend to develop in shallower regions with lower soil strength. As a result, the difference between *β* obtained under stationary RF and RF type 1 is more significant in the reverse dip slope with ratio = 1:2.

For slope ratio of 1:2, the value of slope reliability index under RF type 2 is smaller than those under stationary RF and RF type 1 at each rotational angle. By contrast, for slope ratio of 1:1, the values of slope reliability index under RF type 2 are larger than those under stationary RF and RF type 1 when the rotational angle of bedding < - 30° (i.e., reverse dip slopes). Meanwhile, it would be smaller than those under stationary RF and RF type 1 when the rotational angle of bedding > - 30° (i.e., dip slopes). For steeper slopes with ratio = 1:1, the slip surfaces tend to be shallower [35]. In this case, shallower slip surfaces are more likely to form under stationary RF and RF type 1, and the failure patterns will not be significantly different, as shown in Fig. 9. For RF type 2, in reverse-dip slopes, the slip surface will pass through multiple bedding planes with various soil strengths, and deeper slip surfaces are more likely to develop passing through layers with higher soil strengths. By contrast, for RF type 2 in dip slopes, the slip surface will form roughly along the shallow bedding plane with weak soil strength, which results in the smallest *β.*

In summary, considering rotated transverse anisotropy of soils, the reliability indices of sandy slopes would change insignificantly with major autocorrelation distance, while they would change significantly with minor autocorrelation distance. In addition, the difference between the values of reliability index obtained under the stationary RF and RF type 1 is slight for dip-slopes and the reverse-dip slopes with ratio of 1:1. However, for the reverse-dip slopes with ratio of 1:2, the difference is significant. Moreover, for dip slopes, the results of reliability index under RF type 2 are smallest. By contrast, for reverse-dip slopes, when the slope ratio = 1:1, the results of reliability index under RF type 2 are higher than those under stationary RF and RF type 1.

## Conclusion

This study investigates the reliability of sandy slopes considering anisotropic spatial variation of soils and two non-stationary RFs (depth-dependent, RF type 1, and bedding orientation-dependent, RF type 2). Key findings are presented as follows:

� For horizontal transverse anisotropy:

1. Reliability indices remain stable with increasing major autocorrelation distance but decrease with increasing minor autocorrelation distance, which is consistent with the observations in clay slopes.
2. The non-stationary RF yields results close to those under stationary RF, which is inconsistent with the behavior of clay slopes observed in the previous studies.

� For rotated transverse anisotropy:

1. For dip slopes and reverse-dip slopes with ratio = 1:1, the differences between *β* obtained under stationary RF and RF type 1 are negligible. This observation is inconsistent with that for clay slopes, where the values of *β* obtained under stationary RF are generally significantly higher than those obtained under RF type 1 for all the slope scenarios.
2. RF type 2 yields the lowest values of *β* for dip slopes and reverse-dip slopes with ratio = 1:2, but it yields the highest *β* for reverse-dip slopes with ratio = 1:1.

The findings of this study provide theoretical guidance for the stability assessment and design of sandy slopes in practical engineering. For sandy slopes with horizontal bedding, it is found that the non-stationary characteristics of the spatial distribution of soil parameters have relatively small impacts on slope stability. Therefore, in the engineering practice of designing such slopes, practitioners can appropriately reduce the investment costs related to this issue according to the actual conditions. For instance, in site investigation stages, the investigation resources targeting non-stationary characteristics could be reduced. However, for sandy slopes with inclined bedding, it is crucial to clearly identify the direction of soil strength growth trend in engineering practice. This is because the increasing trends of soil strength along the depth direction or the direction perpendicular to bedding result in significantly different safety margins of a sandy slope with inclined bedding.

## Supporting information

S1 File S1 Data for Figs. 1(a) and (b).S2 Data for Figs. 1(c) and (d).S3 Data for Figs. 1(e) and (f).S4 Data for Fig. 4(a).S5 Data for Fig. 4(b).S6 Data for Fig. 5(a).S7 Data for Fig. 5(b).S8 Data for Fig. 7(a).S9 Data for Fig. 7(b).S10 Data for Fig. 8(a).S11 Data for Fig. 8(b).S12 Data for Fig. 10(a).S13 Data for Fig. 10(b).S14 Data for Fig. 11(a).S15 Data for Fig. 11(b).S16 Data for Fig. 12(a).S17 Data for Fig. 12(b).
(ZIP)
